# Applying lessons from the Ebola vaccine experience for SARS-CoV-2 and other epidemic pathogens

**DOI:** 10.1038/s41541-020-0204-7

**Published:** 2020-06-15

**Authors:** Jayanthi Wolf, Samantha Bruno, Michael Eichberg, Risat Jannat, Sharon Rudo, Susan VanRheenen, Beth-Ann Coller

**Affiliations:** Merck & Co., Inc., Kenilworth, NJ USA

**Keywords:** Drug regulation, Drug development

## Abstract

The world is experiencing an unprecedented global pandemic of coronavirus disease 2019 (COVID-19) caused by a novel coronavirus, Severe Acute Respiratory Syndrome-coronavirus-2 (SARS-CoV-2). Development of new vaccines and therapeutics are important to achieve long-term prevention and control of the virus. Experience gained in the development of vaccines for Ebola virus disease provide important lessons in the regulatory, clinical, and manufacturing process that can be applied to SARS-CoV-2 and other epidemic pathogens. This report outlines the main lessons learned by Merck Sharp & Dohme Corp., a subsidiary of Merck & Co., Inc., Kenilworth, NJ, USA (MSD) during development of an Ebola Zaire vaccine (ERVEBO®) and looks ahead to critical lessons beyond vaccine development. It highlights focus areas for public-private partnership and regulatory harmonization that can be directly applied to current vaccine development efforts for SARS-CoV-2, while drawing attention to the need for parallel consideration of issues beyond development that are equally important to achieve global preparedness and response goals.

## Introduction

First discovered in the Democratic Republic of the Congo in 1976, the highly lethal Ebola virus has infected people in a number of African countries leading to sporadic outbreaks of Ebola virus disease over the past 40 years. While most of the outbreaks have been limited in geographic scope and number of cases, two of the outbreaks over the last 6 years have been large, resulting in major loss of life and socioeconomic disruption in the region. In early 2014, an outbreak of Ebola virus disease caused by *Zaire ebolavirus* started in West Africa and was declared a Public Health Emergency of International Concern in August of that year. By the time the outbreak ended in 2016, more than 11,000 people had died and more than 28,000 people were infected primarily in three countries, Guinea, Liberia, and Sierra Leone^1^.

In the decade prior to the West African outbreak, research efforts for biodefense purposes resulted in the identification of some promising Ebola vaccine candidates that protected monkeys from a lethal challenge of wild-type Ebola virus^2^. These vaccine candidates were not taken into clinical development prior to the West African outbreak for several reasons, which included the inability to demonstrate clinical efficacy in the absence of an ongoing outbreak and lack of interest by the public health and vaccine development community to invest in the lengthy and costly process of vaccine development without a clear demand for an Ebola vaccine^2^. The West African outbreak changed this perspective and numerous vaccines entered clinical development during the outbreak resulting in the generation of safety and immunogenicity data for many of these novel vaccine candidates and the demonstration of efficacy for one vaccine^3^.

## Development of ERVEBO® (*Ebola Zaire Vaccine, Live*), a vaccine for the prevention of *Zaire ebolavirus* disease

**Fig. 1:**
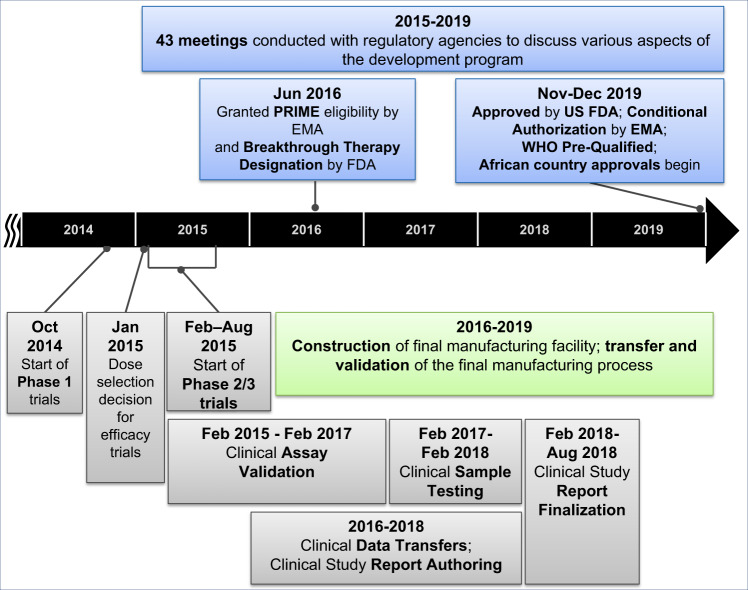
Timeline of the development of the Ebola vaccine from the start of clinical development through approval. Key clinical and manufacturing activities that were essential for the regulatory approval of the vaccine are noted in this figure.

**Fig. 2:**
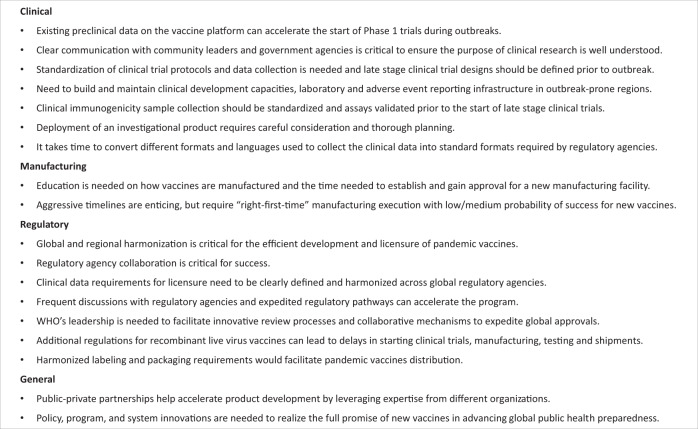
A summary of the lessons learned in pandemic vaccine development. These key points are based on the learnings from the Ebola vaccine program described in this manuscript.

ERVEBO® is a live, attenuated, recombinant vesicular stomatitis virus (rVSV)-based, chimeric-vector vaccine, where the VSV envelope G protein was deleted and replaced by inserting only the envelope glycoprotein of *Zaire ebolavirus*. Extraordinary efforts were made to advance this vaccine candidate through Phase 1, 2, and 3 clinical trials and the data generated in the context of the West African Ebola outbreak has supported its licensure by the US Food and Drug Administration (FDA), conditional authorization by the European Medicines Agency (EMA) and several African countries, along with prequalification by the WHO. The period of 5 years from the start of Phase 1 trials in Oct 2014 to the approval of this vaccine in Nov 2019, was much faster than the typical 10–15 year timeline for vaccine development and approval^4^. A timeline of the key activities in the development of this Ebola vaccine is summarized in Fig. 1 and described in detail in the following sections of this article. Through this Ebola vaccine development effort a number of learnings have been identified, which are highly relevant for the current vaccine development efforts in response to the COVID-19 pandemic. A summary of the key lessons learned can be found in Fig. 2.

## A strong public-private partnership is needed to bring any vaccine candidate through development during the midst of an infectious disease outbreak

This Ebola vaccine was initially designed as a biodefense vaccine by scientists at the Public Health Agency of Canada (PHAC). Preclinical studies performed by PHAC and their collaborators demonstrated that a single-dose of the vaccine candidate was highly efficacious in animal models. In preparation for potential clinical studies or emergency use, PHAC partnered with IDT Biologika to prepare GMP supplies. Beginning in the autumn of 2014, a diverse set of public-private partners mobilized to collaborate on the evaluation and development of the vaccine candidate. The global collaboration spanned a broad range of organizations, including national governments (such as Canada, the United States, and African countries); various agencies (such as the PHAC, US National Institutes of Health, US Centers for Disease Control and Protection, US Biomedical Advanced Research and Development Authority, US Defense Threat Reduction Agency); field response and non-governmental service organizations (such as Medecins Sans Frontieres); global public health entities (such as the World Health Organization, Wellcome Trust, and Gavi); Universities (e.g., the University of Geneva, Dalhousie University) and private sector companies (such as NewLink Genetics, IDT Biologika, and MSD).

Each of these entities had a specific role in the broad partnership, including conducting preclinical studies, manufacturing Good Manufacturing Practices (GMP) trial materials, conducting clinical trials, and funding portions of the research and development. MSD has a well-established history in manufacturing and clinical development of vaccines, with experience in taking novel vaccines through the regulatory process to approval and providing global access. However, MSD did not have prior experience with Ebola virus nor clinical development expertise in the African countries in which the Ebola outbreak was taking place. Therefore, MSD relied on the expertise of organizations that were already involved in the public health response efforts to implement and complete nearly all the clinical trials. Important lessons from this effort are that leveraging the expertise of different organizations can help to accelerate product development and that broad public-private partnerships require strong leadership and coordination to facilitate cooperation of diverse partners with different expertise and missions.

Furthermore, concerns over evaluation of any investigational product are present in less developed parts of the world and these concerns may be further exacerbated during an outbreak^5^. Therefore, clear and transparent communication with community leaders and the community at large, ethical review committees, local ministries of health, and other government agencies is critical to ensure that the purpose of the research is well understood and aligned with accepted local and international norms. Failure to do so can result in delays or roadblocks to research on potentially life-saving products.

## Role of the marketing authorization holder

Ultimately the responsibility to convert the data generated by all the collaborators into a coherent regulatory submission to support licensure of a product lies with the Marketing Authorization Holder. Recognition and proactive planning for that eventuality can help to accelerate the regulatory submission to support product approval. Examples of these types of activities include the following.

### Data integration

MSD’s role in advancing the product to licensure included integrating all the data generated by partners to support the marketing authorization applications. Nine of 13 nonclinical studies, 1 of 2 clinical assay validations, and 11 of 12 clinical trials were performed by partners. MSD needed to obtain all the datasets and reports from these partners to include in the license applications. Data from clinical trials that were performed by partners needed to be migrated into a central database that could be used for data integration, analysis, report-writing, and submission by MSD to regulatory agencies. A lesson learned during this process is to account for the time needed to convert different formats and languages used to collect the data into standard formats required by regulatory agencies, such as use of the Medical Dictionary for Regulatory Activities (MedDRA) and Study Data Tabulation Model (SDTM).

### Clinical trial designs

With the clinical trials all being designed and implemented in the context of the outbreak, it was not possible to harmonize the study designs. While this is not an issue a priori for the conduct of the trials, it introduced challenges when assembling the data to support licensure. For example, it was not possible to conduct extensive integration of the safety data for the product because the data collected and methods used were different for each trial. A key lesson learned during this process is that standardization of clinical trial protocols and data collection is preferred, and that to the extent possible late stage clinical trial designs should be defined prior to outbreaks. Another challenge specifically related to conducting trials in response to emerging diseases is that there is a need to build clinical development capacities, laboratory and adverse event reporting infrastructure in outbreak-prone regions. This was a challenge taken on by individual trial sponsors in the affected countries and required significant and sustained support by the trial sponsors and their local partners. Establishing mechanisms to maintain the capabilities established during outbreak response to provide experienced clinical trial sites for future research is another key lesson learned. Examples of this in action include the ongoing partnership between the US and Liberia and the Partnership for Research on Ebola VACcination (PREVAC) consortium established in the wake of the West Africa outbreak.

### Clinical assays

A key immunogenicity endpoint assay was developed and validated through a collaborative effort by private and public sector partners. The intent was to provide an assay available to all vaccine developers, which might ultimately allow comparison of different vaccine candidates. While the collaborative effort was successful, balancing the input of numerous partners took time. Consequently, clinical assay validation took two years to complete, resulting in a back-log of samples from clinical trials that needed to be processed. A lesson learned is that whenever possible clinical immunogenicity sample collection should be standardized and assays should be validated prior to the start of late stage clinical trials. This requires knowledge of the target pathogen well in advance of clinical trials, which is a challenge for SARS-CoV-2.

### Expanded access use of the vaccine prior to approval

With the rapid collection of efficacy data in support of the vaccine, it was recognized that there was the possibility that new Ebola outbreak(s) could occur ahead of the licensure and availability of licensed doses and that a mechanism was needed to support access to the investigational product. For this scenario, it was necessary to manufacture doses to support outbreak response and establish regulatory frameworks needed to support vaccine deployment (e.g., Compassionate Use, Expanded Access, or Emergency Use). The WHO, MSF, and other groups took the lead in implementing expanded access protocols in African countries, working closely with national governments and ministries of health. Through this effort, more than 300,000 people were vaccinated during the 2018–2020 Ebola outbreak in the Democratic Republic of the Congo^6^. A lesson learned is that the deployment of an investigational product requires careful consideration and thorough planning.

### Manufacturing scaleup

The manufacturing process had to be rapidly scaled-up to handle the needs for emergency use and expanded access programs, in addition to establishing a final manufacturing facility. The approach taken by MSD was to take the initial manufacturing process that was developed at a contract manufacturing organization, scale it up in a clinical manufacturing facility, and transfer it to a commercial manufacturing facility. Technology transfer and the establishment of a new manufacturing facility has many steps and requires significant time to execute (3–4 years is typical for a new vaccine). There is limited understanding outside of vaccine manufacturers and regulators on the rigorous requirements leading to approval of a vaccine manufacturing facility. This broad lack of understanding can lead to misperceptions of “delays” when in fact timelines are driven by the elements required for licensure. For example, although clinical data were available in 2017–2018, the vaccine could not be approved until the commercial manufacturing site was established due to expectations from regulatory agencies that the manufacturing process needs to be validated at the final manufacturing site. A lesson learned is that education is needed on how vaccines are manufactured and the time needed to establish and gain approval for a new manufacturing facility. This is particularly relevant for the COVID-19 outbreak where all aspects of vaccine development are being accelerated at an extraordinary pace with the goal of being able to produce unprecedented quantities of vaccine to address global needs.

Manufacturing site selection for vaccines is a complex decision, which is further complicated when seeking to move fast with incomplete information. The company needed to manage multiple factors, such as existing space, technical capability, infrastructure, and capacity. Site selection also needed to account for the regulations in the country in which the manufacturing site is located and the permits and licenses necessary to support the efforts. The country’s employment environment and labor laws can also impact the ability to hire qualified staff quickly where these factors need to be considered early. A lesson learned is that aggressive timelines are enticing for public health partners, but also require “right-first-time” execution to qualify the facility and manufacturing process, for which the probability of success for a new vaccine is likely medium to low. All parties involved must work to balance the desire to be ambitious (e.g., rapid development timelines) with execution realities and stakeholder expectations. Parallel work and extensive collaboration between manufacturers will be needed in order to successfully bring a SARS-CoV-2 vaccine to the world.

## Global and regional harmonization is critical for efficient development and licensure of pandemic vaccines

### Regulatory agency collaboration is critical for success

From the start of the West African Ebola outbreak, the US FDA, EMA, and Health Canada worked closely with each other and with the National Regulatory Authorities of the impacted West African countries, sharing information about candidate vaccines that were being tested and reviewing the clinical protocols, available data, and benefit-risk profiles. Frequent conversations with manufacturers helped the agencies expedite the start of clinical trials simultaneously in different countries. A lesson learned is that existing preclinical data and availability of clinical supplies afforded the opportunity to start Phase 1 trials rapidly during the West African Ebola outbreak.

### Clinical data requirements for licensure need to be clearly defined

Placebo-controlled, randomized, double-blind studies are typically used to demonstrate the efficacy and safety for new vaccines. However, in the midst of the Ebola outbreak, some countries considered it unethical to administer placebo to at-risk individuals. Different study designs were implemented for the Phase 2/3 trials and a cluster-randomized trial conducted by the World Health Organization in Guinea was the only trial that demonstrated efficacy^7^. Efficacy data from this trial, together with safety data from 12 trials and immunogenicity data with validated assays, were accepted for registration by regulatory agencies. A lesson learned is that the regulatory pathway to licensure for pandemic vaccines need to be clearly defined. Novel trial designs might need to be implemented in order to maximize the possibility of evaluating efficacy during a waning outbreak, and alternative endpoints, such as using immune responses as a surrogate for efficacy, could be used to gain accelerated approval or conditional marketing authorization.

### Frequent regulatory agency interactions are important

Based primarily on interim clinical efficacy data, the manufacturer applied for and was granted access to expedited regulatory pathways, such as Priority Medicines (PRIME) status from EMA and Breakthrough Therapy Designation (BTD) from FDA, which helped to obtain alignment on data requirements for product approval using frequent meetings. Under BTD, the FDA accepted rolling submissions of portions of the Biologics License Application (BLA), which resulted in approval in December 2019, three months before FDA’s target approval date. The FDA determined that a program specific advisory committee meeting was not needed. Similar frequent discussions with EMA under PRIME status, enabled the company to gain alignment on key aspects of the development program quickly.

### WHO leadership is needed to obtain rapid global approvals

WHO is a recognized global public health leader with regional offices in many countries, and established connections with the African Vaccines Regulatory Forum (AVAREF). In order to accelerate vaccine access to African countries, the World Health Organization’s Prequalification Team (WHO-PQT) in collaboration with the EMA and AVAREF developed an innovative facilitated process (roadmap) for decision making on the acceptability of the vaccine for registration^8^. This allowed the company to make simultaneous submissions to EMA, WHO-PQT, and regulatory authorities in 14 African countries, with EMA acting as the reference agency. Following submission, the WHO-PQT and members from AVAREF, which represented the 14 African countries, were invited to participate in teleconferences between MSD and EMA and in the inspection of the manufacturing facility. After a positive opinion from EMA in October 2019, the European Commission granted a conditional Marketing Authorization on November 11th. Within 36 h, the WHO granted prequalification. One month later, African countries started to approve ERVEBO®, starting with Burundi and the Democratic Republic of the Congo. A lesson learned is that strong collaboration and willingness to share information with everyone involved resulted in registration nearly-simultaneously in the countries that needed the Ebola vaccine. For SARS-CoV-2 vaccines, regulatory agencies should prepare for the simultaneous approval of candidates in multiple countries. WHO’s leadership will be needed to facilitate innovative review processes and collaborative mechanisms to expedite approvals.

### Recombinant viral vaccines need to overcome additional regulatory hurdles

Since this Ebola vaccine was a recombinant virus, some countries required a detailed environmental risk assessment prior to the start of clinical trials and as part of the marketing authorization application. In some countries, the vaccine was considered a Bio-Safety Level 2 organism, requiring special handling and permits for the manufacturing and testing sites. These permits took a significant amount of time to obtain, which prevented rapid transfers of material. Samples and technology, which even included parts of the marketing authorization application, required licenses for export because this vaccine is made from the genetic sequence of two viruses that are considered “dual-use” agents and subject to trade controls. A lesson learned is that these regulations led to delays and additional costs incurred in starting clinical trials, manufacturing, testing, and shipments. For manufacturers of SARS-CoV-2 vaccines, countries should consider waiving these requirements, or waiving the fees and expediting these processes.

### Harmonized labeling and packaging requirements would facilitate pandemic vaccines distribution

After approval, companies face challenges with product distribution due to the need to follow country-specific product labeling regulations covering package inserts, cartons, and artwork in addition to serialization requirements. This causes a supply chain issue for a vaccine product intended to be placed in a stockpile and diverted quickly to any country. A lesson learned is that these heterogenous requirements counter the goal of flexibility, speed, and cost-efficiency relevant to emergency preparedness, and there is an urgent need for harmonized solutions. Electronic labeling and Quick Response (QR) codes could be a possible solution, but in most countries, legislation does not currently exist to support this option. There is an urgent need to find a solution to this problem for pandemic vaccines.

## Beyond vaccine development

In addition to considering lessons related to vaccine development for epidemic preparedness, it is equally important to consider the unique challenges and opportunities related to sustainable manufacturing, supply, access, and delivery of these vaccines at the scale and speed needed to achieve preparedness and response goals. Such challenges include right-sizing manufacturing capacity and production while having to accommodate lead-times, managing uncertain demand-and-supply dynamics, ensuring equitable allocation and access, and achieving operational and economic sustainability for all partners. Producing and supplying any vaccine is inherently complex. It is exponentially more complex when there are high levels of uncertainty and unpredictability across nearly every dimension of the program: unpredictable disease, unpredictable and relatively low demand, unpredictable stakeholders and customers, unpredictable timing of need, and unpredictable geographies in which needs might arise. Policy, program, and system innovation—in parallel to research and development innovation—needs to be addressed to realize the full promise of innovative, new vaccines in advancing global public health preparedness.

## Conclusions

Emergency preparedness and response vaccines, such as ERVEBO®, are critical tools in the arsenal against pathogens that can cause pandemics. Public-private partnerships are a powerful and effective approach to develop these vaccines. Setting clear roles, expectations, and accountability enables each partner to bring their respective strengths to the effort. Shared purpose, trust, flexibility, and cooperation are critical to ensure success.

As highlighted in this report and in another recent article^9^, the variability and complexity in regulatory processes and requirements interfere with the speed, flexibility, and efficiency needed to prepare for and respond to public health emergencies. There is an urgent need for global regulatory harmonization and standardization in the approach to the development of vaccines for emergency preparedness. Regulatory agencies need to work together to remove roadblocks and put solutions in place to allow the rapid global development of vaccines for SARS-CoV-2 and other emerging pathogens.
